# Metabolite activity in the anterior cingulate cortex during a painful stimulus using functional MRS

**DOI:** 10.1038/s41598-020-76263-3

**Published:** 2020-11-05

**Authors:** J. Archibald, E. L. MacMillan, C. Graf, P. Kozlowski, C. Laule, J. L. K. Kramer

**Affiliations:** 1 International Collaboration on Repair Discoveries (ICORD), University of British Columbia, Vancouver, Canada; 2 Department of Experimental Medicine, University of British Columbia, Vancouver, Canada; 3 Department of Anesthesiology, Pharmacology and Therapeutics, Faculty of Medicine, University of British Columbia, Vancouver, Canada; 4 Djavad Mowafaghian Center for Brain Health (DMCH), Vancouver, Canada; 5 Hughill Center, Vancouver, Canada; 6 Department of Radiology, University of British Columbia, Vancouver, Canada; 7 ImageTech Lab, Simon Fraser University, Surrey, Canada; 8 Philips Healthcare Canada, Markham, Canada; 9 Department of Physics and Astronomy, University of British Columbia, Vancouver, Canada; 10 UBC MRI Research Centre, University of British Columbia, Vancouver, Canada; 11 Pathology and Laboratory Medicine, University of British Columbia, Vancouver, Canada

**Keywords:** Neuroscience, Physiology

## Abstract

To understand neurochemical brain responses to pain, proton magnetic resonance spectroscopy (^1^H-MRS) is used in humans in vivo to examine various metabolites. Recent MRS investigations have adopted a functional approach, where acquisitions of MRS are performed over time to track task-related changes. Previous studies suggest glutamate is of primary interest, as it may play a role during cortical processing of noxious stimuli. The objective of this study was to examine the metabolic effect (i.e., glutamate) in the anterior cingulate cortex during noxious stimulation using fMRS. The analysis addressed changes in glutamate and glutamate + glutamine (Glx) associated with the onset of pain, and the degree by which fluctuations in metabolites corresponded with continuous pain outcomes. Results suggest healthy participants undergoing tonic noxious stimulation demonstrated increased concentrations of glutamate and Glx at the onset of pain. Subsequent reports of pain were not accompanied by corresponding changes in glutamate of Glx concentrations. An exploratory analysis on sex revealed large effect size changes in glutamate at pain onset in female participants, compared with medium-sized effects in male participants. We propose a role for glutamate in the ACC related to the detection of a noxious stimulus.

## Introduction

Non-invasive neuroimaging techniques are widely applied to gain a deeper understanding of pain in health and disease^1–3^. These approaches primarily focus on function by way of acquiring electroencephalography (EEG)^4–8^, magnetoencephalography (MEG)^5,9,10^, and functional magnetic resonance imaging (fMRI)^11–14^, and have yielded unprecedented insights into the temporal and spatial representation of pain in the brain. Despite major advances, knowledge regarding central mechanisms of pain is incomplete, highlighting a major need to develop techniques suitable for application in humans.

Since the late 1980s, magnetic resonance spectroscopy (MRS) has been employed as a non-invasive tool to quantify various metabolite levels in vivo in the brain, including n-acetyl-aspartate (NAA), total creatine (tCr), myo-inositol (mI), glutamate, and glutamine^15^. More recent investigations have adopted a “functional” approach, whereby acquisitions of MRS are performed in series, over time, to track task-related changes in metabolite levels^16–23^. The theoretical advantage of functional MRS is the potential to more directly measure local events and underlying metabolic processes^16,19,22,24^. This addresses a well-known limitation of conventional neuroimaging techniques, which reflect global activations and deactivations without a direct link to the underlying metabolic processes. In fMRS there is an intriguing opportunity to gain new insights into mechanisms underlying the processing of pain in the brain.

Several corresponding lines of evidence suggest that glutamate is a candidate biomarker for pain^19,22,25–32^. In addition to previous fMRS studies showcasing increased glutamate in response to acutely painful stimulation^16,33–35^, concentrations are altered in patient populations with chronic pain^22,36–38^. Emerging evidence also suggests that pharmacological approaches targeting glutamate receptors are effective in relieving acute^39^ and chronic pain^30,39,40^. Glutamine is another intriguing candidate. Synthesized from glutamate, glutamine is produced in the astrocytes and is an important, intermediary component of metabolism (i.e., glutamate-glutamine neurotransmission cycle)^41^. Unfortunately, the similarity of chemical structures results in overlapping ^1^H NMR spectra at 3 T^41^. As the separation of these is unreliable, the sum (referred to as Glx) can be quantified with higher accuracy^41^.

Among brain regions widely ascribed a role in pain^11,42–45^, the anterior cingulate cortex (ACC) is ideally positioned and composed to acquire high-quality fMRS data. This relates to voxel placement away from air/tissue interfaces and bone (i.e., scalp)^15^ and high gray matter concentration to detect metabolites characterized by low signal to noise (e.g., glutamate)^46^. Seminal studies applying fMRS have yielded mixed results with regards to the metabolic effect of noxious stimulation in the ACC, including no change^47–50^ or increased glutamate and Glx levels^16,21,33,34,51^.

To examine glutamate in the ACC during noxious stimulation, we performed a study in healthy subjects using fMRS. A model of pain was used to generate tonic heat pain, during which participants rated their perceived intensity. Our analysis addressed changes in glutamate and Glx associated with the onset of pain, and the degree by which fluctuations in metabolites corresponded with continuous pain outcomes. We hypothesized that glutamate concentrations in the ACC would increase and track ratings throughout the continuous presentation of tonic heat, reflecting a prominent role in processing pain.

## Methods

### Subjects

Eighteen healthy participants (9 F/9 M, mean age = 26.28, SD = 3.68, range = 21–36 years) were recruited to our study. Each provided informed consent and completed a general health questionnaire. The experimental protocol was explained to each participant in detail before testing. All procedures conformed to the Declaration of Helsinki and were approved by the Research Ethics Board of the University of British Columbia.

### MR experiments

**Figure 1:**
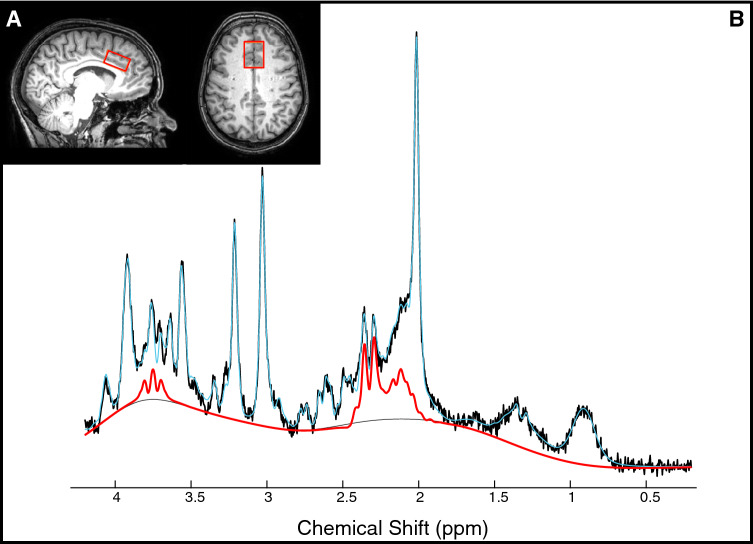
Glutamate levels in the anterior cingulate cortex (ACC). (**A**) Location of MRS voxel in the ACC. (**B**) Example spectrum with raw data (black), LCModel fit (blue), and the contribution of glutamate determined by LCModel (shown in red).

**Figure 2:**
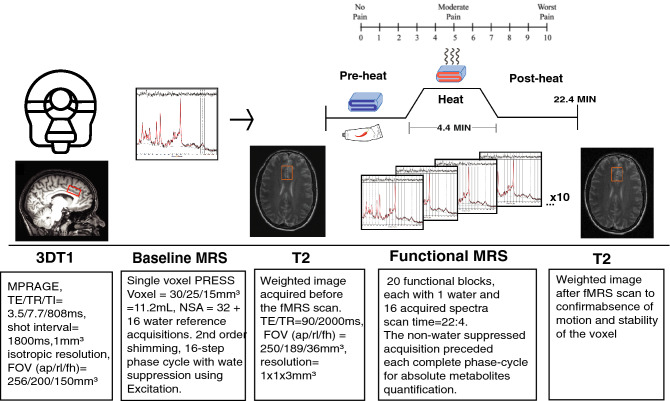
MR data acquisition and pain intervention. The functional scan was uninterrupted for 22.4 min. Participants provided pain ratings via an MRI compatible clicker every 2 min. T_2_-weighted images were acquired before and after the functional MRS scan series to confirm stability of voxel placement by absence of subject motion.

Data were collected using a 3 T Philips Achieva scanner (Best, Netherlands) with a single-channel Transmit-Receive (T/R) head coil. Participants’ heads were immobilized using foam wedges. Sequences included:

(1) *3D T1* (MPRAGE, TE/TR/TI = 3.5/7.7/808 ms, shot interval = 1800 ms, 1 mm^3^ isotropic resolution, FOV (ap/rl/fh) = 256/200/150 mm^3^, scan time = 5:47).

(2) ^*1*^*H-MRS* (PRESS, baseline: TE/TR = 22/4000 ms, NSA = 32, scan time = 3:12, and 16 non-water suppressed spectra were acquired; functional: TE/TR = 22/4000 ms, NSA = 16, scan time = 22:4; ACC, voxel size = 30/25/15 mm^3^ = 11.2 mL, 2nd order shimming, 16-step phase cycle with water suppression using the Excitation option– a Philips variant of CHESS^52^; a non-water suppressed acquisition preceded each complete phase-cycle for absolute metabolite quantification^41,53^ and water signal monitoring, Fig. 1A).

(3) *T*_*2*_*-weighted* (TE/TR = 90/2000 ms, FOV (ap/rl/fh) = 250/189/36 mm^3^, resolution = 1 × 1 × 3 mm^3^, scan time = 0.32 s) acquired before and after the fMRS scan to examine for possible subject motion and confirm stability of voxel placement. (See Fig. 2 for summary).

### Pain stimulation paradigm

To reduce novelty effects associated with our model of experimental pain in the scanner subjects underwent a familiarization period one week prior to acquiring MR data. The same experimental pain procedures (outlined below) performed during MRS acquisition were performed in a laboratory setting.

**Figure 3:**
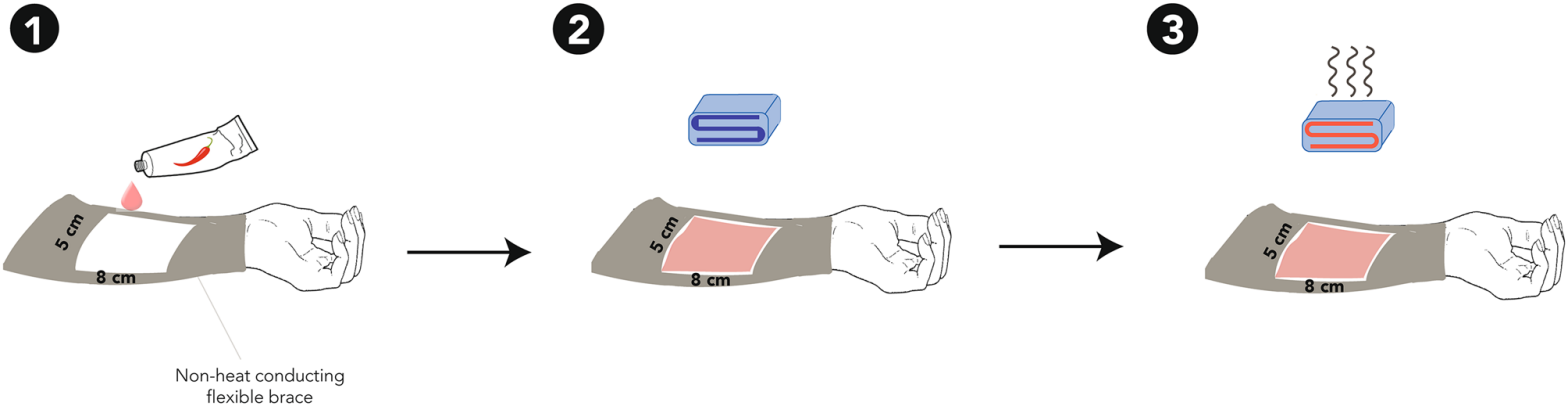
In-scanner pain model. (1) Capsaicin (0.075) was applied on the skin. (2) An inactive thermo-pad was placed on the skin covering the area where capsaicin was applied. 3) The thermo-pad was water-activated (~ 41 °C).

The pain model was developed in preliminary testing and consisted of the application of 0.075% topical capsaicin and heat activation via thermo-pad on the volar surface of the right forearm (~ 8 × 5 cm). Capsaicin was applied immediately after baseline acquisition of MRS alongside a neutral thermo-pad without removing participants from the scanner. The thermo-pad was fixed to the skin and covered the area where capsaicin was applied, and activated by the influx of hot water via plastic tubing conveyed via the penetration panel. A non-heat conducting flexible brace was used to fasten the position of the thermo-pad (Fig. 3). The functional MRS followed by acquiring 16 shots every 1.08 min (total = 22.4 min). After 9 min, the thermo-pad was activated by circulating heated water to reach a temperature of approximately 41 °C at the forearm. Capsaicin was heat-activated for a period of 4.4 min (“heat”). Functional MRS was acquired continuously during heat application and for the reminder of the scan after the heat was removed (i.e., “post heat”).

### Pain intensity ratings

Participants were visually prompted for a pain intensity rating (using the 0–10 numeric rating scale [NRS-11]) every 2 min during the functional MRS scan. Participants indicated an increase, decrease, or no change in perceived pain via an MRI compatible clicker. Feedback was provided by Presentation software (www.neurobs.com/presentation), allowing for the collection of pain ratings throughout the fMRS acquisition while avoiding verbal communication with subjects and head motion. Before commencing with the experimental procedure, participants were familiarized with the rating device.

### Data pre-processing and quantification of metabolites

3D T1 data was segmented into white matter, gray matter and cerebrospinal fluid using FSL BET and FAST^54^. Individual FIDs were pre-processed (eddy current correction, phase correction, frequency alignment, and visual inspection- for quality assurance) in MATLAB (R2016b) using in-house code. 32 shots were averaged for each 2-min block, yielding a total of 11 spectra analyzed using LCModel (v6.3-1H) (Fig. 1B). The simulated basis set was obtained from Steven Provencher specific for the echo time and field strength of this study. LCModel fitting was optimized between 0.2 and 4.2 ppm. The corresponding interleaved non-water suppressed spectra and each participant’s brain tissue water volumes were used to calculate the concentration of NAA, tCr, glutamate and Glx in millimolar (mM)^53,55–57^. Individual water FID’s were fitted to a single-exponential decay curve to extrapolate the water amplitude and the FID decay rate (T2*). This analysis was performed to assure the water amplitude and decay constants were unaffected by BOLD effects.

### Statistical analysis

As a first step, a paired t-test was performed to determine changes in NAA, tCr, glutamate and Glx at the onset of pain. Pain onset was defined as a NRS score ≥ 2 and was compared to the preceding concentration. To assess the specificity of glutamate and Glx, changes in NAA and tCr concentrations were also examined. Water amplitude and T2* stability during rest and pain onset were examined via a paired t-test and effect sizes were calculated. A Pearson correlation was performed between the change in glutamate and Glx concentrations (rest vs pain-onset) and the corresponding pain ratings. An exploratory analysis examined the effects of sex with paired t-tests. Effect sizes were calculated using Cohen’s d^58^ (small effect: d = 0.2, medium effect: d = 0.5, large effect: d = 0.8).

A linear mixed effects model was performed (using the R package lme4 Bates, Maechler & Bolker, 2012) to determine the relationship between glutamate or Glx concentrations and pain intensity ratings over the entire duration of the scan. The statistical model included random slopes and intercepts, as well as the fixed effect of pain ratings [$Model=lmer\left(\left[Glutamate\right]or[Glx]\sim PainRating+\left(PainRating|Subject\right)\right)]$. All statistical tests were performed at an α level of 0.05 in R (version 1.1.442, R Core Team 2012).

## Results

**Figure 4:**
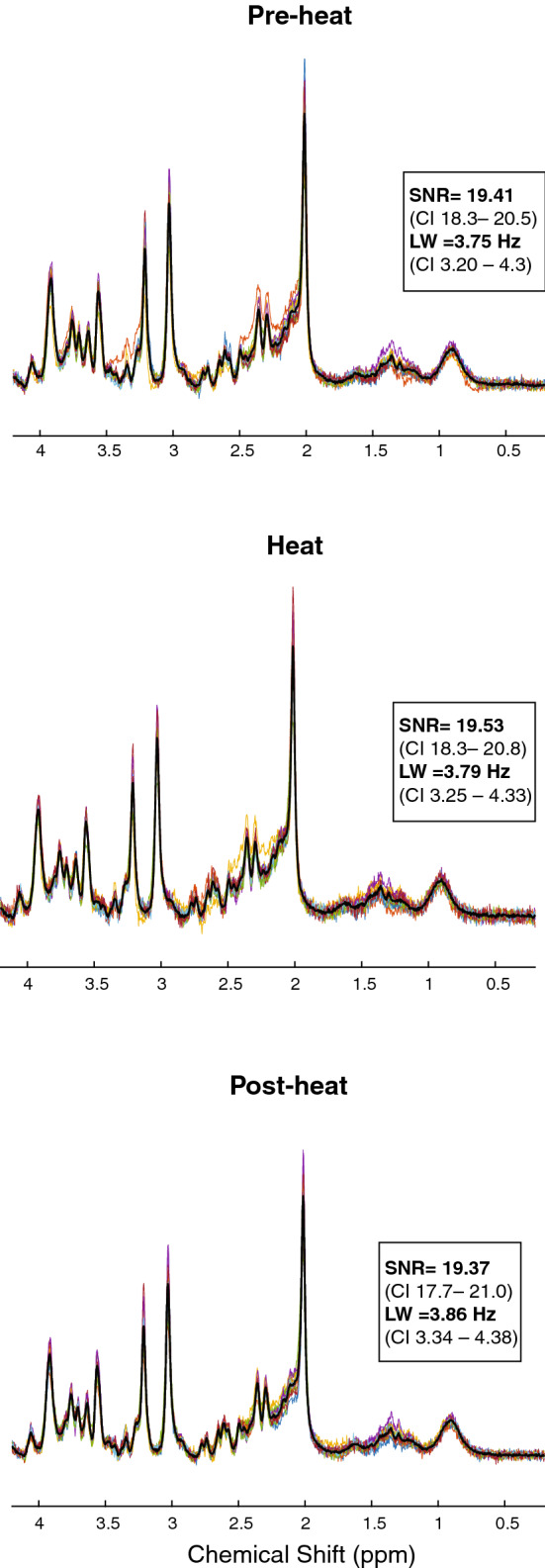
Overview of individual (coloured lines) and mean (black line) baseline-subtracted spectra of included data (n = 15). The functional MRS scan series had 3 different condition (8-min pre-heat, 4-min heat, 8-min post-heat). A consistent high spectral quality was evidenced by high signal to noise ratio (SNR), and narrow line width (LW).

**Figure 5:**
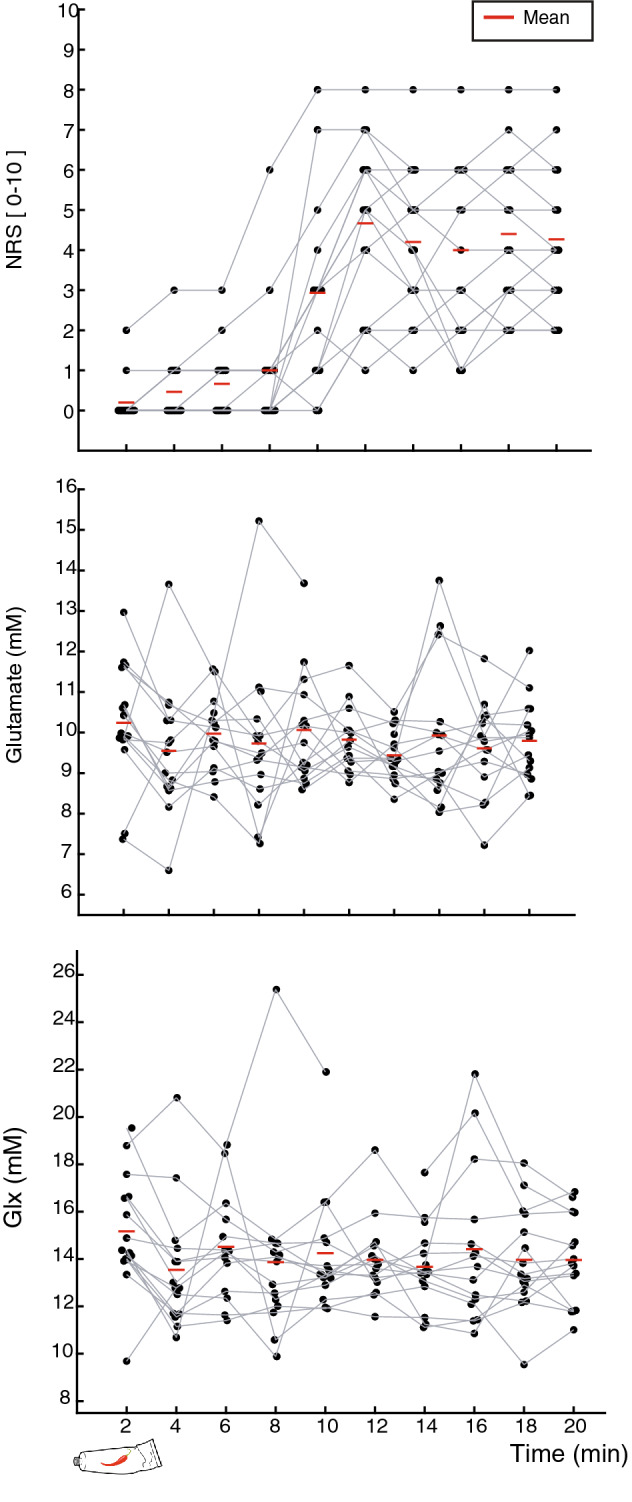
Pain intensity ratings (NRS) and glutamate and Glx concentrations. Values during the functional scan (n = 15).

One participant (f) that underwent the familiarization phase was unable to be scanned due to MR incompatibilities (metallic implant). Data from two participants (1 m; 1 f) was rejected due to excessive motion in the scanner. For the fifteen remaining subjects, SNR was adequate (19.54, 95% CI 18.24–20.84) and linewidth and mean error estimate of glutamate fit were low (3.80 Hz**,** 95% CI 3.20–4.42; 4%**,** 95% CI 3.99–4.00, respectively). Tissue measures were individually estimated for metabolite quantification (Across subjects (mean, SD): WM = 0.27 ± 0.06 range = 0.20–0.45; GM = 0.59 ± 0.04 range = 0.50–0.66; CSF = 0.12 ± 0.05 range = 0.04–0.23). In two subjects, 1 MRS acquisition time point had to be excluded due to excessive motion, as evidenced by a large shot to shot variations in the NAA peak frequency location and linewidth. Figure 4 represents baseline-subtracted spectra quality parameters for the 3 different conditions within the functional scan to illustrate the quality metrics remained the constant throughout testing. Table 1 summarizes average metabolites for baseline and fMRS scans. The average reported pain intensity rating during heat was 3.8 ± 2.0, and 4.2 ± 1.9 during post-heat. Individual pain ratings, glutamate and Glx concentrations during the 20-min fMRS are illustrated in Fig. 5.

**Table 1:** Mean MRS functional metabolite values in the Anterior Cingulate Cortex (± CRLB mM).

fMRS											
Mean (mM)	Baseline	2	4	6	8	10 (heat)	12 (heat)	14	16	18	20
tCr	7.65 ± 0.15	8.07 ± 0.16	7.69 ± 0.15	7.79 ± 0.16	7.63 ± 0.15	7.54 ± 0.15	7.62 ± 0.15	7.26 ± 0.14	7.81 ± 0.16	7.56 ± 0.15	7.67 ± 0.15
Glu	9.90 ± 0.42	10.23 ± 0.46	9.54 ± 0.43	9.97 ± 0.46	9.73 ± 0.43	10.06 ± 0.42	9.82 ± 0.43	9.43 ± 0.42	9.92 ± 0.42	9.61 ± 0.41	9.79 ± 0.41
Glx	14.06 ± 0.55	15.17 ± 0.59	13.54 ± 0.52	14.51 ± 0.56	13.86 ± 0.56	14.24 ± 0.55	13.96 ± 0.54	13.67 ± 0.52	14.41 ± 0.53	13.96 ± 0.53	13.95 ± 0.55
NAA	9.41 ± 0.26	10.03 ± 0.28	9.60 ± 0.26	9.68 ± 0.26	9.71 ± 0.25	9.48 ± 0.26	9.49 ± 0.26	9.05 ± 0.25	9.71 ± 0.26	9.52 ± 0.24	9.58 ± 0.26

*CRLB* Cramer-Rao minimum lower bounds, *tCr* total creatine; *Glu* glutamate; *Glx* glutamate + Glutamine; *NAA* *N*-Acetyl aspartic acid.

### Neurochemical concentrations at pain onset

**Figure 6:**
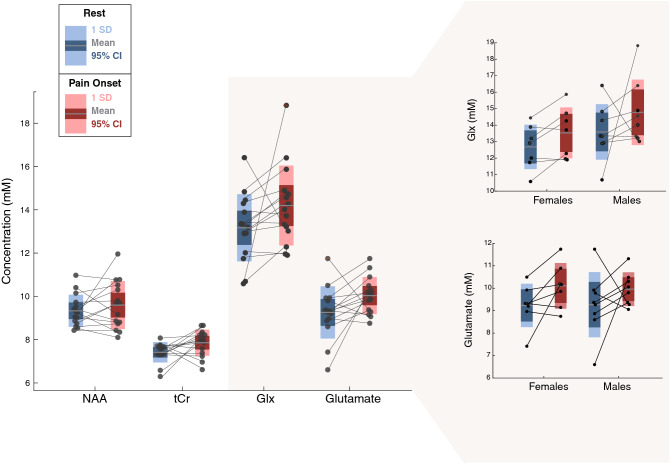
NAA, tCr, Glx and Glu ACC concentrations during rest (no pain perception) and pain onset for each volunteer (n = 15). Exploratory sex-based analysis showed a large effect for change in glutamate at pain onset in females (Cohen’s d = 0.88, CI − 0.43 to 2.21, n = 7), compared with a medium sized effect for males (Cohen’s d = 0.60 CI − 0.57 to 1.73, n = 8). Changes in Glx at pain onset were medium sized for both men (Cohen’s d = 0.65 CI − 0.53 to 1.83) and women (Cohen’s d = 0.58 CI − 0.69 to 1.87). There was no difference in pain rating at onset between men and women (t = − 0.24, p-value = 0.80 CI − 1.2 to 0.9).

Glutamate and Glx concentrations both showed similar sizes in percent increases at the onset of pain (Glutamate: + 8.38%, t = 2.04, p = 0.06; Glx: + 7.25%, t = 1.74, p = 0.11) (Fig. 6). The change in glutamate and Glx correspond to large to medium effect sizes (Cohen’s d_glutamate_ = 0.74 CI − 0.05 to 1.55; Cohen’s d_Glx_ = 0.60 CI = − 0.18 to 1.40). There was no significant correlation between the change in glutamate or Glx concentrations and pain ratings (rest vs. pain onset) (Glutamate: r = − 0.36, p = 0.18; Glx: r = − 0.22, p = 0.42). tCr concentrations tended to increase at the onset of pain (+ 6.0%, t = 2.06, p = 0.05; Cohen’s d_tCr_ = 0.83 CI = 0.02–1.64) (Fig. 6). NAA concentrations were unchanged from the onset of pain compared to rest (+ 2.81%, t = 0.9, p = 0.34; Cohen’s d_NAA_ = 0.28 CI − 0.49 to 1.05). There was no significant change in the water amplitude (-3.0%, t = 1.45, p = 0.17, Cohen’s d = 0.18) nor T2* (0.36%, t = − 0.42, p = 0.67, Cohen’s d = − 0.02) values during rest and pain onset.

An exploratory analysis of sex revealed there was no difference in glutamate and Glx concentrations at rest (Glutamate: t = − 0.05, p-value = 0.95, CI − 1.39 to 1.32, Cohen’s d = 0.02, CI − 1.22 to 1.17; Glx: t = − 1.17, p-value = 0.26, CI − 2.58 to 0.76, Cohen’s d = 0.59 CI − 1.82 to 0.63), between males (Glutamate = 9.2 ± 1.4; Glx = 13.5 ± 1.6) and females (Glutamate = 9.2 ± 0.9; Glx = 12.6 ± 1.3). When examining changes at pain onset, large effect sizes were seen for glutamate in female participants (+ 8.63%, Cohen’s d_female_ = 0.88, CI − 0.43 to 2.21), compared with medium sized effects in male participants (+ 7.45%, Cohen’s d_male_ = 0.60 CI − 0.57 to 1.73). Medium effect sizes are seen in Glx for both males and females (+ 6.26% Cohen’s d_female_ = 0.58, CI − 0.69 to 1.87; + 8.04%, Cohen’s d_male_ = 0.65, CI − 0.53 to 1.83) (Fig. 6). At pain onset, the average NRS pain score was 2.9 ± 0.9 across all subjects, and was not significantly different for females (3.0 ± 0.8) compared to males (2.8 ± 1.1; t = − 0.24, p-value = 0.80, CI − 1.2 to 0.9).

### Neurochemical concentrations and pain intensity ratings

**Figure 7:**
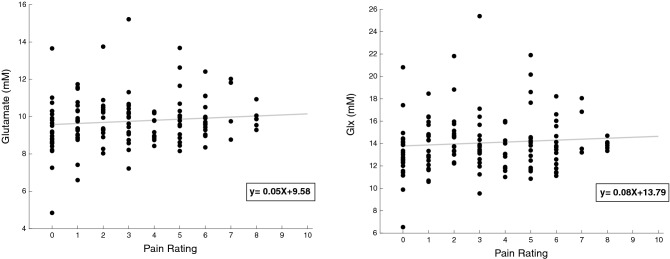
Linear mixed effects model during the 20 min fMRS scan. There was no relationship within subjects over the entire duration of data acquisition between pain rating and glutamate or Glx.

Based on a linear mixed effects analysis, glutamate and Glx concentrations were not significantly correlated with pain intensity ratings within subjects across the acquisition of fMRS (Glutamate: ß = 0.059 standard error (SE) = 0.06, p = 0.39; Glx: ß = 0.08 SE = 0.12, p = 0.48) (Fig. 7). This suggests that, overall, neither glutamate nor Glx concentrations modulated in relation to the perception of pain over the entire duration of the 20-min functional scan. NAA and tCr were also not related to pain over the duration of our experiment (NAA: ß = -0.05 standard error (SE) = 0.04, p = 0.24; tCr: ß = -0.01 SE = 0.05, p = 0.83).

## Discussion

In the current study, the relationship between glutamate and Glx in the ACC and perception of noxious heat stimulation was examined using fMRS. Medium to large effect sizes were detected for increases in glutamate, Glx, and tCr at the transition to pain (i.e., pain onset, when NRS pain score ≥ 2 for the first time). NAA, but not creatine remained stable at the onset of pain. There was no overall relationship between pain ratings and glutamate or Glx concentrations, indicating the transient nature of increased concentrations. Our observations suggest a role for glutamate in the ACC in initially detecting but not tracking pain.

### Glutamate and Glx concentrations changes in the ACC

Evidence of regional activation in the ACC in response to noxious stimulation dates back to seminal studies applying fMRI and PET^27,59–61^. These are further supported by observations from single neuron recordings, which first raised the notion that a region of the ACC was selective for pain^62,63^. Subsequent investigations have demonstrated a more complex role, with the ACC, critically involved in “salience detection”^64,65^ and affective dimensions of pain^59^. On the basis that the ACC represents a prominent area of the “neurological signature of pain” responsive to analgesic interventions^11,66,67^, it remains a high priority area of study for pain researchers.

Five studies have previously investigated changes in glutamate and Glx in the ACC during the presentation of a noxious stimulus^16,21,47–49^, of which two have observed an increase in Glx^16,21^ and glutamate^16^. Similar to Mullins et.al, we applied a long-duration noxious stimulus and observed a similar percent increase in glutamate compared to rest (+ 8.3% compared to Mullins et al. + 9.4%). To our knowledge, ours is the first study to distinguish the onset of pain from tonic appraisal. Based on our results, glutamatergic activation turns “on” independent of the intensity and “off” in the presence of a stimulus that remains painful. The lack of prolonged increases in glutamate concentrations contrasts that overserved for fMRI in various brain regions, including the mid-ACC, where BOLD remains elevated for the duration of the painful stimulation^68^. In agreement with our observations, a transient increase in extracellular glutamate in the ACC^69^ and elsewhere in the CNS (e.g., spinal cord^25,28,70–75^) has been reported in animal models using microdialysis following noxious stimulation induced by way of formalin injection. Critically, in response to formalin, the second wave of pain behaviour is not matched with an increase in extracellular glutamate, as is observed for the initial wave of pain behaviour. Collectively, this points to the glutamatergic response to noxious stimulation, captured by way of fMRS, representing one of the numerous “other” roles of the ACC in appraising sensory stimuli^64,65,76,77^.

Mechanisms underlying the tendency for glutamate to increase in response to noxious stimulation are, at this time, poorly understood. Glutamatergic synapses play a role in sensory transmission, including pain^25,26,28,78–80^. In the ACC, all major forms of ionotropic and metabotropic glutamate receptors have been isolated^81,82^. Metabolically, glutamate is stored as glutamine in astrocytes, and the balanced cycling between these two neurochemical is essential for normal functioning of brain cells^83^. In addition to functioning as a neurotransmitter, glutamate also serves as a metabolic precursor^83^. Glutamate and glutamine are compartmentalized in neurons and glia, respectively^84^ and are directly connected to neurotransmission and energy metabolism^85,86^.

Preliminary evidence suggests that glutamate levels may reflect neurotransmission^19^. One practical example is that NMDA antagonists significantly and paradoxically *increase* glutamate levels in the brain^40^. This could be explained for by NMDA receptor antagonists acting on post-synaptic neurons, preventing binding of glutamate, which increases the visibility of glutamate molecules to MRS (i.e., molecular tumbling rates). Available evidence suggests that glutamate in different locations in the neuron can be more or less visible to MRS depending on acquisition parameters^87^. A longer echo time (TE > 15 ms) is more sensitive to compartmental shift (i.e., neural activity), compared to tightly packed glutamate in presynaptic vesicles^19,87^. The increased sensitivity of a change in compartmentation is due to a slower T_2_ relaxation rate—the rate at which MRS signal decays over time, as glutamate is freely floating (i.e., not packed in the vesicle). This theory is highlighted by a recent meta-analysis comparing fMRS studies with short and long echo times; with short echo time experiments demonstrating smaller increases in glutamate in response to sensory stimulation compared to longer echo times (~ 2.7 versus 6.4%)^19^. Our study used an echo time of 22 ms. Based on the aforementioned theory, the MRS signal may be more sensitive to glutamate levels moving from the presynaptic neuron to the postsynaptic neuron (i.e., compartmental shift).

### Other metabolites

Our analysis focused on examining glutamate and Glx. Other metabolites such as NAA and tCr, which have more prominent resonances, were examined as a reference, to establish the specificity of changes in glutamate in response to pain. NAA was selected from other potential metabolites because turnover is slow, and is not responsive to acute metabolic disturbances^41^ and as expected, concentrations remained stable, even at the onset of pain. Creatine, interestingly, increased in response to pain and did so similarly to glutamate and Glx. A previous study also reported a dynamic tCr response following noxious stimulation albeit a decrease^51^. Fluctuations in creatine concentrations are a major concern because tCr levels are commonly used as a reference in H-MRS (i.e., creatine ratios)^88^, including in functional studies^21,34,47,50^. While the essential role of creatine in energy metabolism and cell energetics is well-established^89^, emerging evidence suggests a role in neuromodulation and neurotransmission^89–93^. The mechanisms underlying changes in tCr in response to pain require further elucidation, our observations, as well as those previously published^51^, raise concerns about the functional stability of creatine in task-related MRS studies.

### Sex effects

In an exploratory secondary analysis, we investigated the effect of sex on changes in glutamate and Glx in response to pain. This analysis reflects a high priority area in pain research^94^, and a commitment to understanding the role of sex in biomedical research^95^. Various neuroimaging techniques have been employed to probe sex differences in the brain, revealing prominent variations in anatomy and function^96^. However, recent meta-research has suggested that a reporting bias may overestimate the magnitude of sex-related differences in the brain^97^. With regards to MRS, sex-related differences in resting glutamate levels have been reported in various brain areas (e.g., hippocampus) but not in the ACC^98^. To our knowledge, studies applying noxious stimulation during the acquisition of fMRS have not previously examined or reported differences related to sex. Our results confirm similar resting glutamate and Glx concentrations in the ACC between men and women and nominally larger increases in glutamate and Glx in response to pain in women. Sex differences in glutamatergic signalling have also been reported in animal models^99^, with female rats tending to be more sensitive to NMDA receptor modulation (e.g. Ketamine)^100–102^. Further fMRS research should incorporate sex-based analyses to gain a better understanding of glutamatergic signalling in humans.

### Limitations and future directions

The most notable limitation of our study a small sample size (n = 15). To account for this, we have reported the results of conventional significance testing, as well as effect sizes for all relevant comparisons. Future studies should include a larger sample of men and women to further distinguish the role of excitation in the ACC in response to pain. Another limitation of our study is that we did not acquire GABA edited spectra. Previous fMRS studies have demonstrated the capacity to acquire estimates of GABA^21,50^, one of which has demonstrated reduced concentrations following the presentation of a noxious stimulus^21^. Reduced GABA concentrations are in agreement with our observations, insofar as this reflects increased excitation. Finally, the field of MRS is rapidly evolving, and new methods of acquisition have emerged. Recent guidelines suggest the localization sequence semi-LASER offers reduced localization error (reducing chemical shift displacement) compared to PRESS^15^. The incorporation of simulated metabolite basis sets into the routine analysis is recommended for capturing the full spectral detail from short TE acquisitions^15^, future studies should aim to incorporate these suggestions.

## Conclusion

In summary, healthy participants undergoing tonic noxious stimulation demonstrated increased concentrations of glutamate and Glx at the onset of pain. Subsequent reports of pain were not accompanied by increased glutamate or Glx concentrations. We propose a role for glutamate in ACC related to the detection of a noxious stimulus.

## Data Availability

The datasets generated during and/or analysed during the current study are available from the corresponding author on reasonable request.
